# Unravelling the genetic basis of Fusarium seedling rot resistance in the MAGIC maize population: novel targets for breeding

**DOI:** 10.1038/s41598-019-42248-0

**Published:** 2019-04-05

**Authors:** Popi Septiani, Alessandra Lanubile, Lorenzo Stagnati, Matteo Busconi, Hilde Nelissen, Mario Enrico Pè, Matteo Dell’Acqua, Adriano Marocco

**Affiliations:** 1 Institute of Life Sciences, Scuola Superiore Sant’Anna, Pisa, 56127 Italy; 2 Department of Sustainable Crop Production, Università Cattolica del Sacro Cuore, Piacenza, 29122 Italy; 3 Department of Plant Biotechnology and Bioinformatics, Ghent University, Ghent, B-9052 Belgium; 4 VIB Centre for Plant Systems Biology, Ghent, B-9052 Belgium

## Abstract

Fungal infection by *Fusarium verticillioides* is cause of prevalent maize disease leading to substantial reductions in yield and grain quality worldwide. Maize resistance to the fungus may occur at different developmental stages, from seedling to maturity. The breeding of resistant maize genotypes may take advantage of the identification of quantitative trait loci (QTL) responsible for disease resistance already commenced at seedling level. The Multi-parent Advance Generation Intercross (MAGIC) population was used to conduct high-definition QTL mapping for Fusarium seedling rot (FSR) resistance using rolled towel assay. Infection severity level, seedling weight and length were measured on 401 MAGIC maize recombinant inbred lines (RILs). QTL mapping was performed on reconstructed RIL haplotypes. One-fifth of the MAGIC RILs were resistant to FSR and 10 QTL were identified. For FSR, two QTL were detected at 2.8 Mb and 241.8 Mb on chromosome 4, and one QTL at 169.6 Mb on chromosome 5. Transcriptomic and sequencing information generated on the MAGIC founder lines was used to guide the identification of eight candidate genes within the identified FSR QTL. We conclude that the rolled towel assay applied to the MAGIC maize population provides a fast and cost-effective method to identify QTL and candidate genes for early resistance to *F*. *verticillioides* in maize.

## Introduction

Maize (*Zea mays* L.) is a key crop for food, feed, and industrial products. It is the cereal species with the highest grain production worldwide, with more than one billion tons harvested each year^1^. Maize production, however, is menaced by numerous pathogens that affect both the quantity and the quality of the grain produced^2–4^. The occurrence of the diseases varies by year and depends on several factors including environment, susceptibility of maize varieties, and cropping practices^5^. In recent years, climate change is causing disease outbreaks even in geographic regions in which they were infrequent in the past^6^.

*Fusarium verticillioides* (Sacc.) Nirenberg is a predominant endophyte and pathogen of maize causing substantial yield losses and reduction of grain quality. Maize can be infected by the fungus at all growth stages, from the early vegetative phases to maturity. The fungus can be transmitted through infected kernels and cause systemic infection that eventually contribute to the development of seedling diseases^7^ including seedling rot^8,9^, root rot and stalk rot^7^. Indeed, in seedling rot, reduction in seed emergence and seedling growth may be observed when seedlings are inoculated with *F*. *verticillioides* strains producing fumonisin B_1_^10,11^. The amount of damage caused by this disease depends on the extent of rotting: if rot is extensive, the embryo may die and the seed will not germinate. Even for seeds that are not severely rotted, germination in unfavorable environments will be slow or absent due to presence of the internal fungus^2^. In many plant-pathogen interactions, the expression of the resistance to pathogens depends on the stage of development at which the plant is infected. Plants are generally more susceptible to diseases in early stages rather than in later stages^12^. Plants already resistant to pathogens at early stages may be able to increase their ability to control infection at later stages^13^. Conversely, genotypes which are susceptible at early stages may either acquire disease resistance or remain susceptible over time^13,14^.

The genetic basis of *F*. *verticillioides* resistance in maize is not yet fully understood. Several studies reported genetic sources of resistance to *F*. *verticillioides* at maturity stage associated to Fusarium ear rot (FER)^15–19^. However, complete resistance to FER has not yet been achieved^20–22^. Resistance to the disease is indeed a quantitative trait under polygenic control^23^. Previous studies reported a number of quantitative trait loci (QTL) associated with FER resistance and identified multiple loci with relatively small effect^17,19,24–26^. At the seedling stage, the evaluation of genetic resistance to Fusarium seedling rot (FSR) may benefit from seed inoculation with *F*. *verticillioides* in laboratory assays under uniform spore concentration standards and inoculation conditions^27,28^. Even if phenotypes derived from this type of inoculation may be distant from the agronomic manifestation of adult plants FER, screening for resistance in early stages allowed the discovery of QTL and candidate genes for kernel and seedling resistance to fungal proliferation^27,28^, adding new insights on the relevant basic biology of the seedling-fungus interaction.

Traditionally, QTL mapping studies utilize recombinant inbred lines (RILs) derived from bi-parental crosses. RIL populations provide high QTL mapping power, as they allow to incorporate pedigree information to track the co-segregation of genetic and phenotypic diversity in a limited set of variation^29,30^. The choice of two founder lines, however, limits the allelic variation used to identify QTL, and fails to leverage the broad diversity available at the species level. Multi-parental population designs emerged in crops to broaden allelic diversity and to increase the recombination frequency in RILs^31^. In maize, a highly diverse nested association mapping (NAM) population has been developed altogether incorporating the diversity of 26 inbred lines^32^, and a multi-parent advance generation intercross (MAGIC) population was developed starting from eight diverse founder lines. These populations provide the means to conduct high-definition QTL mapping on a set of highly diverse RILs exploiting high definition genomic data produced on the founder lines, such as genome sequencing and transcriptomics^33^. In controlled phenotyping experiments such as rolled towel assay (RTA), where a limited amount of RILs is deployed, the MAGIC design may be advantageous, as MAGIC RILs undergo three intermating generations before inbreeding, accumulating recombination events. Moreover, each individual MAGIC RIL is a combination of eight founder haplotypes, increasing the diversity available within each phenotyped MAGIC RIL collection as compared to bi-parental RILs. For these reasons, the MAGIC maize provides high QTL mapping power in lower RIL numbers^33,34^.

The objective of this study was to identify QTL and candidate genes conferring resistance to FSR resistance in the MAGIC maize (MM) population. A panel of 401 MM RILs was phenotyped for FSR, seedling length and weight using the RTA. A broad variation of FSR was observed in the MM population. Ten QTL were discovered underpinning three seedling phenotypes, and candidate genes were identified using transcriptomic and sequencing information. We show that the coupling of advanced genetic resources with phenotyping conducted at the seedling stage provides high definition mapping power and resolution on detecting QTL for resistance to *F*. *verticillioides* infection at seedling stage.

## Results

### Phenotypic analysis of the MAGIC maize population

**Figure 1:**
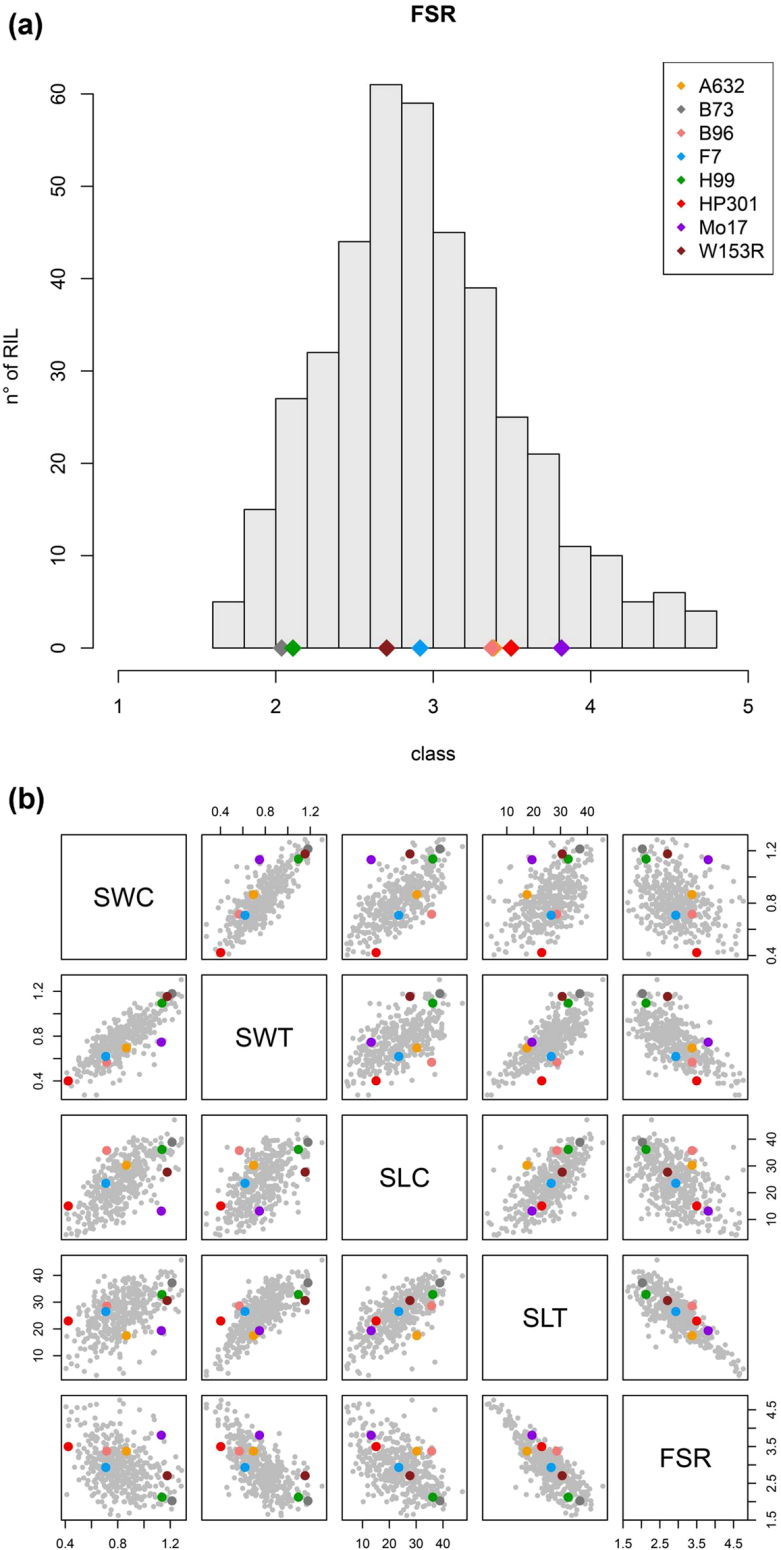
Fusarium seedling rot (FSR) distribution in MM RILs (panel a) and trait correlation (panel b). The coloured dots in the histogram report the severity class of the eight MM founder lines according to legend.

The RTA screening conducted on 401 RILs reported wide phenotypic variation for all the measured traits in the MM population (Table S1). The occurrence of Fusarium seedling rot in control (FSRC) RTA was minimum. FSRC values ranged from 1 to 1.6 (Table S3), showing that the control had low to no presence of *F*. *verticillioides* (Fig. S3). Those few RILs with FSRC scores above 1 may be due to a low level of seed-borne endophytic *F*. *verticillioides* infection inside the RIL seed lot. This is also indicated by the pink fungal mycelium seldom observed around the germinating seeds (Fig. S1). The Fusarium seedling rot in treated rolls (FSR) values followed instead a normal distribution, broader in RILs than in parental lines (Fig. 1). The FSR scores for founder lines ranged from 2 to 3.8 with a mean value of 2.9, while the FSR values for the RILs ranged from 1.6 to 4.7 with a mean value of 2.9 (Table S3). This result confirms the presence of several FSR alleles segregating in the MM population. Among the founder lines, B73 was the most resistant, and Mo17 was the most susceptible relative to other MAGIC founder lines. We observed that 19% of the MM RILs were below FSR class 2, which we consider as resistant. Seedling weight and seedling length also showed a normal distribution in control and treated RTAs (Fig. S3). Mean values of seedling weight in treated rolls (SWT) both in founders and RILs were lower as compared to mean values of seedling weight in control rolls (SWC), indicating that seedling weight decreased after *F*. *verticillioides* inoculation (Table S3). Conversely, mean values of seedling length in treated rolls (SLT) in RILs were slightly increased as compared to mean values of seedling length in control rolls (SLC) (Table S3). We also observed correlations between FSR, seedling length and seedling weight, indicating that a more severe infection reduced weight and length of germinating seeds (Fig. 1b).

A true replication on 36 RILs chosen at random from the full collection was performed to check for consistency of phenotypic data (Table S4). A Pearson correlation coefficient of 0.83 was estimated between FSR scores across RIL replicas. Similarly, correlation for SWT and SLT across replicas was estimated in 0.86 and 0.83, respectively (Fig. S4). An analysis of variance (ANOVA) testing the hypothesis of a difference between FSR values in the two replicas was not significant (F value = 0.332, p value = 0.566), supporting the reliability of the RTA phenotyping method.

### QTL mapping and candidate genes identification

Founder haplotypes in the population were previously reconstructed based on which we performed linkage mapping for the measured traits. The QTL scan detected 10 QTL above the suggestive significant threshold (Table 1). Sequencing data on the MM founder lines was used to identify candidate genes. The RNA sequencing produced on MM founder seedlings without fungal infection, identified altogether nineteen protein coding genes which were differentially expressed (DE) in a constitutive way among founder lines and were located within QTL confidence intervals. Simultaneously, the full sequencing data produced on the MM founder lines was used to refine QTL confidence intervals by conducting association mapping (AM) at the QTL, reporting altogether 359 protein coding genes. For each QTL, its genomic position and the presence of suggestive candidate genes from DE and AM analyses are reported below.

**Table 1:** Identified QTL for the Fusarium seedling rot (FSR) in treated rolled towel assays (RTAs), seedling length (SLT) in treated RTAs and seedling weight in control (SWC) and in treated (SWT) RTAs.

No.	QTL name	Chr	Start pos (Mb)	Peak Pos (Mb)	Stop Pos (Mb)	Peak pos marker	% Trait variation	Phenotype
1	qSWT1.1	1	179.6224	180.0152	188.0503	PZE.101139280	8.2	SWT, SWC
2	qSWC2.1	2	197.1761	198.9759	212.4568	SYN22309	6.2	SWC
3	qSWC2.2	2	204.1674	209.6068	212.4568	PZE.102163621	6.7	SWC
4	qSLT3.1	3	3.826982	4.712836	6.684788	SYN5653	6.6	SLT
5	qSWT3.1	3	6.975485	9.31421	15.065589	PZE.103016459	6.5	SWT
6	qFSR4.1	4	2.118457	2.841088	3.467877	SYN17710	6.7	FSR
7	qFSR4.2	4	240.7926	241.8789	241.7959	PZE.104158108	7.3	FSR, SLT
8	qFSR5.1	5	167.6253	169.6211	172.8555	PZE.105112628	6.7	FSR, SLT, SWT
9	qSWC6.1	6	80.12923	80.30413	82.12695	SYN22588	6.6	SWC
10	qSWT6.1	6	121.2138	124.706	128.4967	PZE.106070356	6.2	SWT

**Figure 2:**
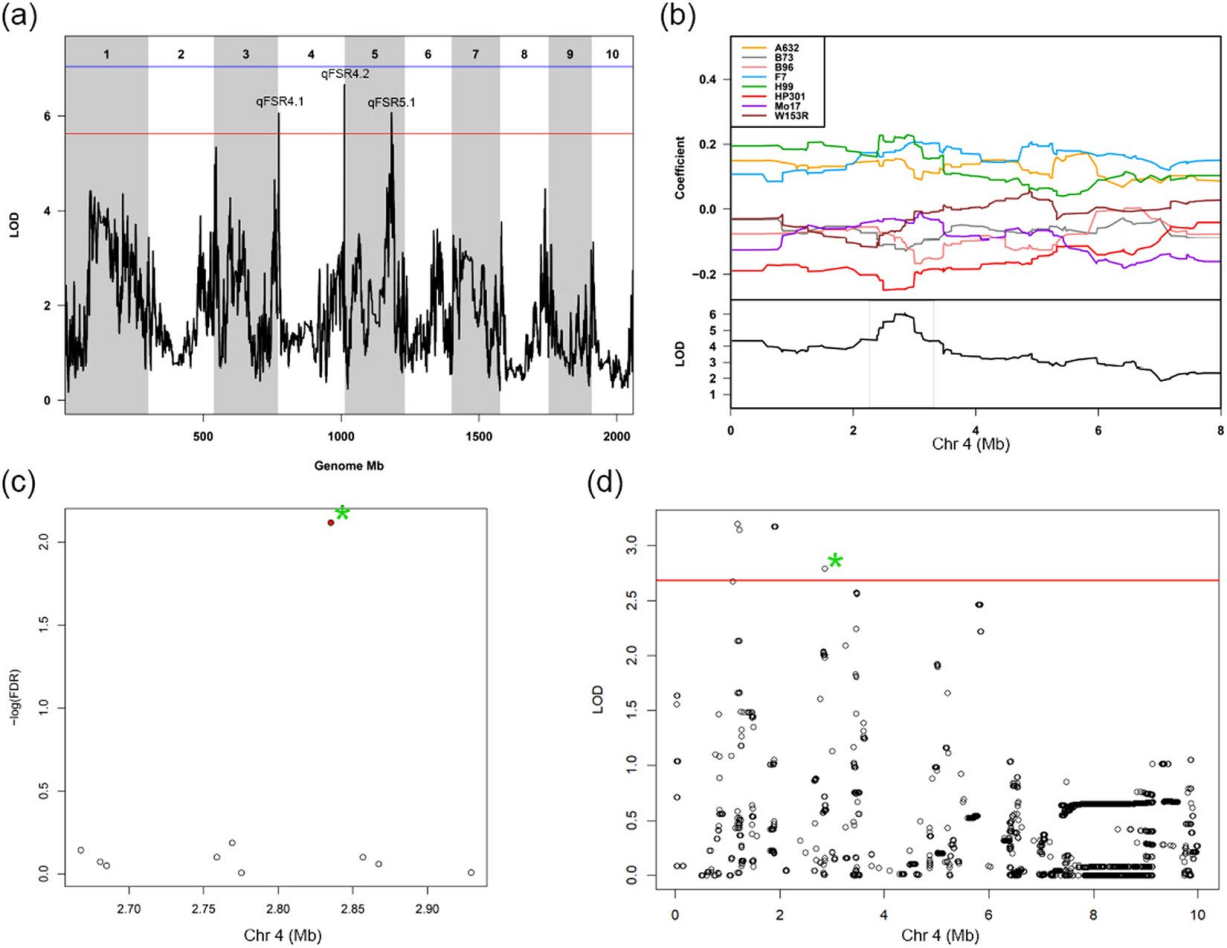
Dissection of the QTL qFSR4.1 for Fusarium seedling rot (FSR) resistance. In panel a, the linkage mapping result shows three QTL, named according to Table 1. On the x axis, the genomic position in Mb, on the y axis the LOD significance of the presence of QTL for FSR. The suggestive QTL contributing to FSR at the beginning of chr 4 is further characterized. In panel b, the upper plot shows the founder allele effects estimated by the QTL mapping model at the qFSR4.1 locus. The founder HP301 contributes with low allele at this QTL. The bottom plot shows a detail of the LOD curve, with a grey frame highlighting the QTL confidence interval. Panel c reports the significance for a test for differential expression of genes matching the founder allele effects within the QTL confidence interval (Mb position on the x axis). Genes are represented by points. The green asterisk highlights the position of *GRMZM2G062129*, whose expression is significantly different among founder lines in agreement with estimated effects at the QTL. Panel d shows the result of association mapping in the QTL area. The red line is the threshold of significant association after 500 permutation (*P* < 0.05). The dots represent the imputed SNPs. The green asterisk highlights a SNP at once significantly associated with the phenotype and in close vicinity with *GRMZM2G062129*.

Fusarium seedling rot in treated RTAs identified three QTL (Table 1). The qFSR4.1, was unique for FSR and mapped on chromosome (chr) 4 with peak position at 2.8 Mb. The qFSR4.1 accounted for 6.7% of FSR variation (Fig. 2a). At this QTL, the estimated founder effects showed that HP301 contributed the resistance allele providing low FSR values, while F7, H99 and A632 contributed the susceptible allele(s) (Fig. 2b). The DE analysis at this locus reported *GRMZM2G062129* as the sole gene matching the founder effects at FDR <0.05 (Fig. 2c; Table S5). This gene encodes for a protein belonging to the family of senescence-associated proteins. The AM approach on the same QTL detected five single nucleotide polymorphism (SNPs) (Fig. 2d) that targeted 19 protein coding genes, including *GRMZM2G062129*, also detected by DE (Table S6).

The qFSR4.2 was identified on chr 4 with peak position at 241.8 Mb and was pleiotropic with SLT (Table 1; Fig. S5a). This QTL explained the highest phenotypic variation for FSR, accounting for 7.3% of the phenotypic variation. According to the estimation of founder allele effect conducted by the QTL mapping model, Mo17 provided low FSR values, while B96 and W153R provided high FSR values at this locus (Fig. S6a). The DE analysis did not report any candidate gene (Table S5), while AM reported 1,300 closely clustered SNPs targeting only 33 protein coding genes (Table S6).

The qFSR5.1 was identified on chr 5 at 169.6 Mb, accounting for 6.7% of FSR variation and pleiotropic with SLT and SWT (Table 1; Fig. S5a). The estimation of the founder allele effect at this locus indicated that H99 contributed to low FSR values, while F7 and A632 allele(s) provided high FSR values (Fig. S6b). No candidate genes were identified by the DE approach (Table S5). The AM analysis detected 482 significant SNPs targeting 76 protein coding genes within the QTL confidence interval (Table S6).

Seven additional QTL specific to SL and SW were identified (Table 1). The SLT reported only one significant peak (qSLT3.1) at 4.7 Mb on chr 3 accounting for 6.6% of phenotypic variation (Table 1: Fig. S5b). At this QTL, HP301 contributed low seedling length values, while W153R, F7, and H99 provided high seedling length (Fig S4c). The DE approach did not identify any candidate genes (Table S5), and AM identified 360 SNPs reporting 27 protein genes (Table S6).

For SWC, we identified three unique QTL. The qSWC2.1 located on chr 2 at 198.9 Mb and accounts for 6.2% of phenotypic variation (Table 1; Fig. S5c). At this locus, the DE approach identified 13 protein coding genes (Table S5) matching founder allele effects, with B96 contributing to low seedling weight, and HP301 providing the opposite effect (Fig. S6d). AM identified 51 SNPs reporting 33 protein coding genes (Table S6). Another QTL for seedling weight in control was qSWC2.2 located on chr 2 at 209.60 Mb explaining 6.7% of phenotypic variation (Table 1; Fig. S5c). This QTL is very close but not overlapping to qSWC2.1, and shows the same founder allele effect pattern (Fig. S6e). The DE approach identified one protein coding gene (Table S5) and AM reported 137 significant SNPs targeting 23 protein coding genes (Table S6). A third SWC QTL, qSWC6.1, was located on chr 6 at 80.3 Mb and accounted for 6.6% of the phenotypic variation (Table 1, Fig. S5c). The founder allele effect estimated that A632 contributed the low seedling weight values while W153R contributed high values (Fig. S6f), but DE analysis did not identify any candidate gene (Table S5) and AM did not reported significant associated SNPs (Table S6).

For SWT we reported three QTL, qSWT1.1 on chr 1 at 180 Mb and pleiotropic with SWC (Table 1; Fig. S5d). Mo17 contributed high phenotypic values at this QTL (Fig. S6g), and DE analysis identified one candidate gene (Table S5), *GRMZM2G132763*, encoding for a BRASSINOSTEROID INSENSITIVE 1-associated receptor kinase 1. The AM identified 2852 significant associated SNPs targeting 119 genes (Table S6) including *GRMZM2G132763* also detected by DE. The qSWT3.1 was located on chr 3 at 9.3 Mb and accounted for 6.5% of phenotypic variation (Table 1; Fig. S5d). The founder allele effect showed that B96 contributed low values of seedling weight while H99 contributed high values (Fig. S6h). DE detected three protein coding genes (Table S5) and AM identified 41 significant associated SNPs targeting 20 protein coding genes (Table S6) in the QTL interval. The third QTL for SWT (qSWT6.1) was located on chr 6 at 124.7 Mb and was responsible for 6.2% of phenotypic variation (Table 1; Fig. S5d). The founder allele effect estimated that B96 provides low values of seedling weight whereas H99 and W153R provided high values (Fig. S6h). The DE approach did not identify any candidate genes (Table S5) while AM reported 59 significant associated SNPs targeting nine protein coding genes (Table S6). No QTL was identified for FSRC and SLC.

## Discussion

In this study, a simple and efficient method of disease screening was applied to a large collection of MM RILs. The RTA allowed us to evaluate resistance to *F*. *verticillioides* infection in controlled conditions in germinating maize kernels, providing consistent visual symptoms of infection easy to score (Fig. S2). The RTA favoured the fungus establishment and growth in a standardized manner, maximizing the infection and making it visible through several symptoms: kernel colonisation, lesions, browning of the coleoptile and reduced seedling growth. Our findings support its utilization to provide valuable information for understanding the molecular basis of FSR. The normal distribution observed for FSR (Fig. 1) is the result of the highly diverse genotypes segregating in the MM population. FSR range and mean are similar in RILs and founder lines, but the presence of transgressive segregation suggests that several resistance alleles are simultaneously segregating in the population. SW and SL also show near-normal distributions (Fig. S3). As expected, these traits are negatively correlated with FSR (Fig. 1b), since *F*. *verticillioides* infection limits germination of maize kernels and hampers seedling growth and vigour.

The QTL mapping on the MM population identified FSR resistance loci explaining a relatively small variation of the phenotype, the highest being 7.3% for qFSR4.2. This result confirms the complexity of the genetic basis of *F*. *verticillioides* resistance, as already reported in previous studies. Giomi *et al*.^26^ reported QTL for ear rot accounting for 5% to 11.9% of the phenotypic variation in a bi-parental population. Another bi-parental population reported FSR QTL explaining up to 12.6%^27^, and GWAS panels representing the maize core diversity and U.S.A. inbred lines reported R^2^ between 3% to 12%^19–22^. In this scenario, a high-definition mapping of sequences underlying disease resistance may be highly beneficial in speeding up the deployment of new varieties with genetic resistance to the disease.

Several QTL associated with *F*. *verticillioides* resistance associated to seed rots^27,28^ and ear rots have been mapped in different maize populations^35^. In this study, two major FSR resistance QTL on the distal region of chr 4 (qFSR4.1 and qFSR4.2) and another major QTL on chr 5 (qFSR5.1) were identified. These two chromosomes are often reported in extant *F*. *verticillioides* resistance literature, although QTL are often mapped in contrasting positions. Previous studies using SSR markers and field phenotyping reported QTL for resistance to FER in bin 4.03, 4.09, and 5.05^17^. A GWAS combined with linkage mapping conducted on a panel of tropical maize inbred lines using SNPs and SSR marker reported FER QTL on chr 4 at 183 Mb and 199 Mb^36^. More recently a FER resistance QTL was identified on bin 4.10 using SSR and SNPs^21^. Similarly, multiple FER resistance QTL were identified on chr 5, at bin 5.03 and bin 5.04^24^, and more recently at bin 5.07^26^. Multiple GWAS efforts reported marker trait associations with FER resistance on chr 4 at 7 Mb, 9 Mb and 124 Mb^19^ and on chr 5 at 30 Mb^22^, 64 Mb^19^, and 16 Mb, 56 Mb, 182 Mb^27^. Similarly, Ju *et al*.^27^ reported marker trait association with Fusarium seed rot on chr 4 at 4.86 Mb and 198 Mb^27^. In this frame, our results confirm that chr 4 and 5 are hot spot regions for *F*. *verticillioides* resistance, even though the location of resistance QTL are not completely overlapping. Different genetic and physical position of the QTL may stem by the use of different genome assemblies as well as by the different genetic materials employed for genetic mapping^37^. In this work, we are using a QTL mapping approach based on the haplotype reconstruction of MM RILs and on Fusarium phenotyping at seedling stage, both features that may contribute to the specificity of our results. The capacity to narrow down QTL mapping intervals to candidate genes, thanks to DE and AM, however, provides the possibility to overcome potentially contrasting findings and to directly access the causal variants of QTL. Indeed, regardless the positional inaccuracies that possibly derive from variation in the quality of local genome assemblies, expression data directly points to candidate genes, reinforcing these findings. The DE is based on RNA sequencing data produced on healthy seedling at the fourth leaf stage^33^. The genes on which DE is based are therefore representing constitutive differences among founder lines rather than transcriptional changes triggered by fungal infection. The expression level of these genes may thus be interpreted as cause, rather than an effect, of the resistance phenotype. The concurrent identification of some candidate genes by both DE and AM reinforces their importance, as these analyses are based on non-overlapping data (RNA sequencing and DNA variants) and methods. It may indeed be the case that SNP variants in regulatory regions affect RNA expression levels, and by that trait levels.

A clear example of the above is the case of qFSR4.1, both AM and DE methods pinpointed *GRMZM2G062129* as a promising candidate gene. According to Plaza Monocot 3.0, the best ortholog of this gene in *Arabidopsis thaliana* is ARABIDOPSIS A-FIFTEEN (*AAF*). In *Arabidopsis* this gene encodes for a senescence-associated protein involved in leaf senescence, a cellular response to oxidative stress and to reactive oxygen species in metabolic processes. The transcriptome analysis of expression profiles in 3-week-old *A*. *thaliana* plants overexpressing *AAF* reported an up-regulation of genes related to senescence, oxidative stress and pathogen defence^38^. Further studies may elucidate the role of this gene in enhancing maize resistance to *F*. *verticillioides*. Within qFSR4.1, the AM approach identified 18 additional candidate genes, among which three are clearly suggestive of disease resistance mechanisms. *GRMZM2G475505* encodes for an osmotin-like protein that belongs to the subclass pathogenesis-related type 5 (PR-5) protein in tobacco whose expression pattern is controlled by ethylene signaling and fungal infection^39^. Recently, a study in *A*. *thaliana* reported an osmotin-like protein involved in defense response to fungal infection of *Aspergillus ochraeus*^40^. The *GRMZM2G311664*, also identified by AM in qFSR4.1, is homologous to the blight resistance protein R1B in potato, where it functions as a pathogen-receptor gene^41^. The *GRMZM2G455321* encodes a protein homolog to an NB-ARC domain-containing disease resistance protein in *A*. *thaliana*, which mediates the responses associated with defense mechanism to fungi^42^. Altogether, these results suggest that this QTL might be a hot spot for resistance in the MM population.

Candidate genes identified from the dissection of qFSR4.2 and qFSR5.1 are also suggestive of disease response mechanisms, specifically in the recognition of pathogen conserved molecules also known as microbe-associated molecular patterns (MAMPs). When MAMPs are recognised by pattern recognition receptors (PRRs) in plant plasma membranes, the defence mechanisms are elicited^43,44^. Two such PRRs were identified by AM in qFSR4.2 and qFSR5.1: *GRMZM2G059671*, encoding a serine/threonine-protein kinase, and *GRMZM2G040964*, encoding a BRASSINOSTEROID INSENSITIVE 1-associated receptor kinase 1 protein. These PRRs were also identified in recent transcriptomic studies on *F*. *verticillioides*-maize interaction^45,46^. These two QTL also yielded two candidate genes encoding heat shock proteins (HSP), *GRMZM2G059851* and *GRMZM2G134806*. HSPs are responsible for protein folding, assembly, translocation and degradation under stress conditions, including pathogen infection^47^.

The RTA allowed us to identify QTL for seedling weight and seedling length in presence of the fungus (Fig. S2). Mostly, previous studies reported QTL and candidate genes for seedling traits by phenotyping root traits associated with nitrogen and water use efficiency^48^, hormone response^49^ or nutrient intake^50^. Our study reported some interesting QTL for seedling traits in infected plants on chr 1 (qSWT1.1) and chr 3 (qSLT3.1). Both QTL have small allelic effects, yet yielded suggestive candidate genes. The AM in qSLT3.1 reported other gene models. *GRMZM2G077197* is homologous to the *A*. *thaliana* gene coding for the regulatory protein NPR1. NPR1 is known as a receptor of salicylic acid, which is an essential hormone in plant immunity^51^. From the QTL associated to seedling weight, we also found two notable candidate genes, *GRMZM2G132763* detected from DE in qSWT1.1 and *GRMZM2G004696* detected from AM in qSWT3.1. The *GRMZM2G132763* is similar to *GRMZM2G040964* from qFSR5.1 that also encodes for the BRASSINOSTEROID INSENSITIVE 1-associated receptor kinase 1, while *GRMZM2G004696* encodes for an auxin-responsive protein and has its best ortholog with *AT4G14550* which encodes the indole-3-acetic acid inducible 14 (IAA14) protein in *A*. *thaliana*. IAA14 is a member of the Aux/IAA protein family involved in lateral root development^52^. Finally, we identified *GRMZM2G161335* as a notable candidate gene from AM in qSWT1.1. This gene encodes a DIMBOA UDP-glucosyltransferase BX9, hence it is involved in benzoxazinoids biosynthesis, secondary metabolites implicated in the chemical defence of cereals^53^. DIMBOA is the major benzoxazinoids expressed in maize seedlings^54^, and researchers reported that it plays a vital role in enhanced disease resistance of mycorrhizal plants of maize against sheath blight^55^.

Overall, the QTL mapped in this study furthers the available knowledge on the genetic basis of *Fusarium* resistance in maize. Additional studies based on phenotyping on larger numbers of MM RILs may provide additional power to confirm these QTL and to identify FSR loci with even smaller allelic effects. It is worth pointing out that, even though only 401 RILs have been phenotyped, the MM provided sufficient power to detect QTL with an effect as small as 6.2% (Table 1). This finding supports the high mapping power provided by the MM genetic background, that couples high minor allele frequency and high density of recombination events^33^. The high-density genotyping data available on the MM, sided by the extensive genomic characterization developed on its founder lines, makes this population a handy tool to be combined with RTA phenotyping for identifying candidate genes for resistance. The transcriptomic characterization of MM founder lines infected during the RTA would provide a finer characterization of the perturbation of maize expression levels in presence of *F*. *verticillioides*. When integrated with QTL mapping data reported in this study, such data could support the identification of candidate resistance genes whose expression is triggered by fungal infection.

Our study focused on the seedling developmental stage, therefore further investigations in MM RILs resistance at maturity is needed to evaluate adult plant resistance to FER. To the best of our knowledge, there is no report associating *F*. *verticillioides* resistance between maize seedling and mature stages. Indeed, the fungal infection in mature plants may involve different mechanisms than those in seedlings, including mechanical wounding promoted by insect species including *Ostrinia nubilalis*^56^, *Busseola fusca*^57^ and *Sesamia nonagrioides*^58^ that favor fungal colonization. Further studies are needed to network *F*. *verticillioides* resistance QTL across different stages of the plant cycle to reinforce the prioritization of candidate genes delivering resistance in the field. We anticipate that we are measuring *F*. *verticillioides* resistance in the MM grown in open field in order to confirming the role of candidate genes identified in the present study. In these respect, the study of *Fusarium* resistance in a hybrid background may also significantly contribute to the production of hybrids with higher field resistance. The RTA bioassay is being applied on recombinant inbred crosses (RIXs) produced by the crossing of MM RILs to detect and study heterotic effects on disease resistance. Altogether, these advanced genetic, statistical, and phenotypic methods bear the potential to speed up the development of genetic resistance to *F*. *verticillioides* in maize.

## Material and Methods

### Genetic material and genotypic data

The MM population was derived from eight genetically diverse maize inbred lines (A632, B73, B96, F7, H99, HP301, Mo17, W153R) crossed in a funnel breeding design^33^. MM RILs are genotyped with 56,110 SNPs markers on the Illumina MaizeSNP50 BeadChip^59^. The MM founder lines have their full genome sequenced and RNA expression levels at the fourth leaf stage, as previously described^33^. In this study, 529 MM RILs, with seeds produced in the same season, were subjected to phenotyping by RTA for FSR resistance and seedling traits. The analyses were restricted to 401 RILs having sufficient germination rate. In the retained RILs, a dynamic filtering method was applied to account for different germination rates of MM RILs and to subtract their effect to trait values (see below).

### Phenotyping

Forty seeds with similar size and without visible damages were selected for each MM RIL and for MM founder lines, 20 to be used as a treated sample and 20 to be used as a control. Seeds were surface-sterilized in a solution of 70% ethanol shaken for 5′ at 50 rpm to reduce seed-borne contaminations. Ethanol was removed and seeds were washed by sterilized bi-distilled water for 1′, then by a commercial bleach solution for 10′, and finally rinsed three times (5′ each) with sterilized bi-distilled water.

Two RTAs, for control and treated conditions, were prepared for each MM RIL. Three towels of germination paper (Anchor Paper, Saint Paul, MN) for each RTA were moistened with sterilized bi-distilled water; twenty seeds were placed evenly spaced on two base towels, and were covered with the third towel. In the treated RTA, the 20 seeds were each inoculated with 100 µl of a 3.5 × 10^6^ ml^−1^ spore suspension of *F*. *verticillioides* ITEM10027 (MPVP 294). The strain was isolated from maize in South Tuscany, Italy, by the Department of Sustainable Crop Production, Piacenza, Italy, and deposited in their fungal collection and also in the Institute of Sciences and Food Production, National Research Council of Bari, Italy (http://server.ispa.cnr.it/ITEM/Collection). The towels were then rolled up, placed vertically in a bucket and kept in groups of five in transparent plastic bags separately for treated and control to avoid cross-contamination. RTAs were incubated at 25 °C in the dark for seven days. In order to test the reproducibility of the RTA method, 36 RILs were randomly sampled and phenotyped in two independent RTA experiments.

After incubation, RTAs were laid on a work bench and opened to phenotype seedlings for FSR, seedling length (SL), and seedling weight (SW). A depiction of an RTA during phenotyping is provided in Fig. S1. All traits were measured on each seedling in the control RTAs, named as FSRC, SLC, SWC and in the treated RTAs, named as FSR, SLT and SWT. The FSR was assessed on each seedling by a visual evaluation of seedling size and visible colonization of *F*. *verticillioides* in a scale from 1 to 5 adapted from previous research on soybean seedlings^60^. On this scale, 1 corresponds to complete resistance and 5 corresponds to complete susceptibility. The full description of the FSR scores is provided in Table S2. A visual example of each FSR score is reported in Fig. S2. Seedling length was determined by measuring the length of the seed from the tip of the shoot to the tip of the root, in centimetres. Seedling weight was determined by measuring the weight of the whole germinated seed using a laboratory scale, in grams.

### Phenotypic data analyses

Data analyses were conducted with R^61^. The phenotypic value for each MM RIL was obtained by averaging trait values of individual seedlings. In order to account for failures in germination that may result in overestimating FSR values, raw phenotypic data was filtered following two criteria. The first criteria determined that MM RILs showing less than 14 germinated seeds (70%) in the control RTA were discarded. The second criteria required that after data cleansing, the phenotypic value deriving from the treated RTA was normalized by germination rate in the control RTA. In fact, it was not possible to discern between a seed which was not germinated because of high severity of the disease from a seed which was not germinated for reasons other than the inoculation of *F*. *verticillioides* conidia. To avoid this bias, for each RIL, the seeds failing to germinate in the control RTA were counted, and an equal number of seeds with FSR class 5 (not germinated) in the treated RTA was discarded. For example, if in the control RTA of a specific RIL there were four seeds which were not germinating, then four seeds with FSR class 5 in the treated RTA were excluded from the averaging of FSR for that RIL. For SL and SW, non-germinated seeds were considered as missing data. The correlation between FSR, seedling length and seedling weight were evaluated. Normality was checked for all phenotypes with R plots.

### QTL mapping

QTL mapping was performed on the probabilistic reconstructions of RIL haplotypes based on high quality SNP^33^. The QTL mapping pipeline on the MM used custom R scripts based on the R package for QTL mapping using diversity outbred mice (DOQTL)^62^. Briefly, founder haplotypes were reconstructed on MM RIL genomes using SNP markers information in a hidden Markov model (HMM). Linkage mapping of QTL was performed with an additive model estimating the eight founder haplotype’s contribution to the tested phenotype. An empirical significance threshold for QTL logarithm of odds (LOD) scores was determined by 500 permutations. Suggestive and high significance thresholds were determined at the 37^th^ and 90^th^ percentile of the permuted LOD scores distribution. Confidence intervals were calculated with a 2 LOD drop from the highest association at each QTL.

Transcriptomic information generated on the founder lines was used to guide the identification of candidate genes within QTL. RNA-seq data was produced from the most basal 0.5 cm of the fourth leaf of each MM founder line grown in the growth chamber under controlled growth conditions (24 °C, 55% relative humidity, 170 μmol m^−2^ s^−1^ photosynthetically active radiation at plant level in a 16 h/8 h day/night cycle)^33^. The transcriptomic data is therefore not specific to fungal infection and allows to target constitutive difference in RNA expression levels among MM founder lines. For each discovered QTL, the mapping model estimates each MM founder effect at the QTL. The expression level of genes falling within ± 1 Mb of the QTL confidence intervals was tested for differential expression (DE) matching founder effects at the QTL. The DE analysis was conducted using a generalized linear model implemented in R/edgeR^63^. A multiple testing correction based on false discovery rate (FDR) with threshold 0.05 was applied using R/qvalue^64^ to adjust the significance of each individual DE test.

The full genome sequence produced on founder lines was used to conduct association mapping (AM) in QTL intervals. The founder genome sequences were imputed on reconstructed RIL haplotypes, contributing with high density SNPs exceeding those generated on the RILs^33^. The SNPs deriving from imputation were used in AM against the same phenotype as the QTL under study, narrowing down the confidence interval to the sole markers surpassing the AM significance threshold at the 95^th^ percentile of the *p-value* distribution derived by 500 permutations of the phenotype. Then, putative genes were searched within a 50 kb interval downstream and upstream of the significant associated SNPs.

The candidate genes obtained from AM and DE analysis on each QTL, according to B73 RefGen_v3 assembly^65^, were assessed for their function in the integrative orthology search PLAZA 3.0^66^ for monocots, and discussed when relevant. Full details of the above methodologies can be found in Dell’Acqua *et al*.^33^.

## Supplementary information

Supplementary info

Table S2

Table S4

Table S5

Table S6
