# Supplementary material for: Unravelling the genetic basis of Fusarium seedling rot resistance in the MAGIC maize population: novel targets for breeding
Source: Sci Rep. 2019 Apr 5;9:5665. doi: 10.1038/s41598-019-42248-0 (PMC6451006; doi:10.1038/s41598-019-42248-0)
Supplement: Supplementary file 1 — Supplementary info [file 41598_2019_42248_MOESM1_ESM.pdf]

SUPPLEMENTARY MATERIALS

TITTLE

Unravelling the genetic basis of Fusarium seedling rot resistance in the MAGIC maize population: novel targets for breeding

AUTHORS

Popi Septiani[1], Alessandra Lanubile[2], Lorenzo Stagnati[2], Matteo Busconi[2], Hilde Nelissen[3,4], Mario Enrico Pè[1], Matteo Dell'Acqua[1], Adriano Marocco[2*]

[1]Institute of Life Sciences, Scuola Superiore Sant'Anna, Pisa, 56127, Italy

[2]Department of Sustainable Crop Production, Università Cattolica del Sacro Cuore, Piacenza, 29122, Italy

[3]Department of Plant Biotechnology and Bioinformatics, Ghent University, Ghent, B-9052, Belgium

[4]VIB Centre for Plant Systems Biology, Ghent, B-9052, Belgium

[*]Corresponding author: adriano.marocco@unicatt.it

(a)

(b)

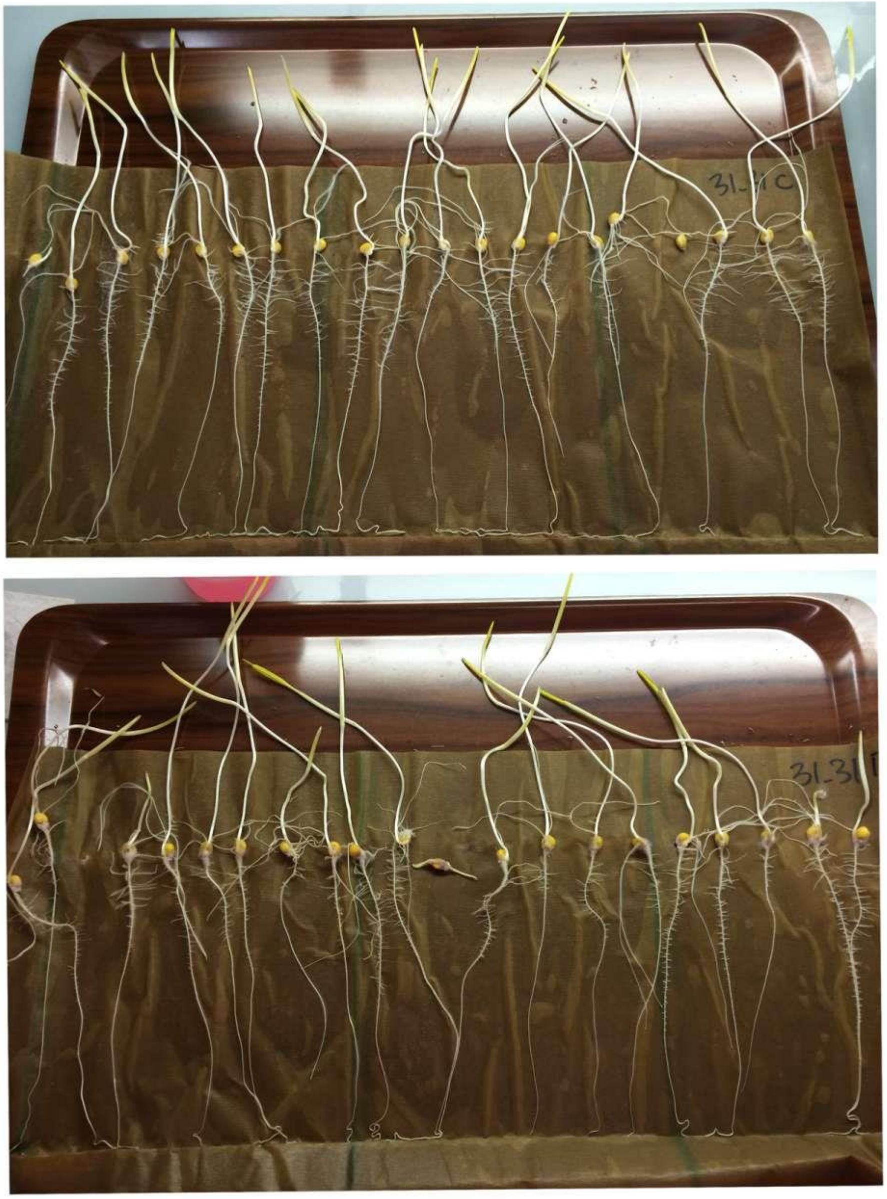

Figure S1. Depiction of rolled towel assay. Panel **a** shows the germinating seeds in control RTA and panel **b** shows the germinating seeds in treated RTA. Severity classes were measured based on the colonization of *Fusarium verticillioides* on the germinating kernels (Table S1).

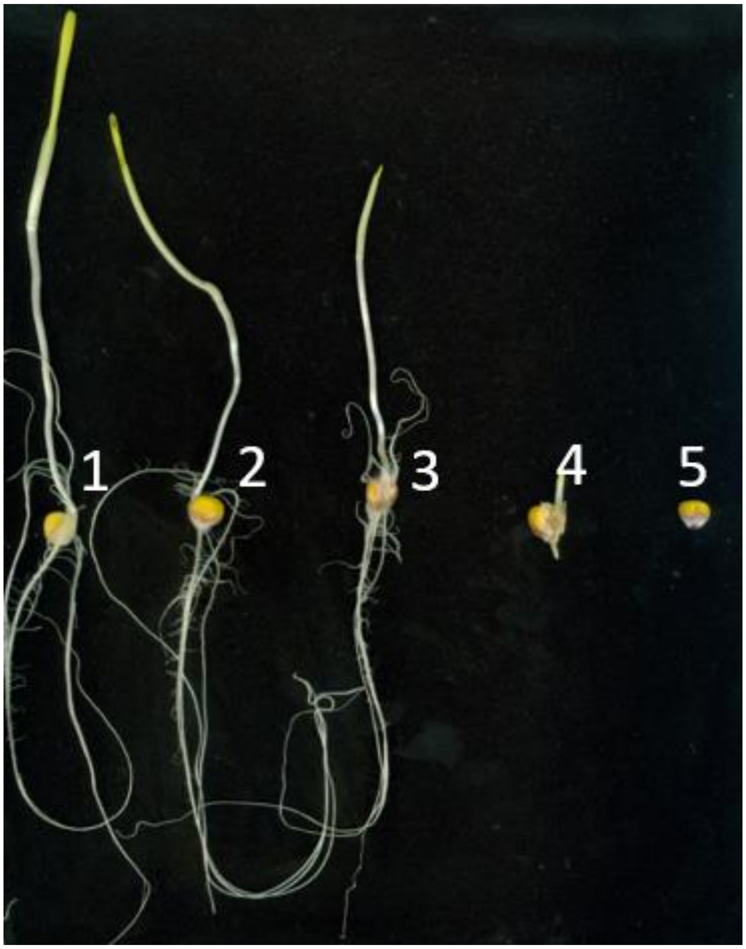

Figure S2. Depiction of each severity score. Seedlings were rated from 1-5 scale as previously reported (Stagnati *et al.,* 2019), where: 1 = healthy, germinated seedlings with no visible signs of colonization; 2 = germination and colonization of the kernel near the pedicel; 3 = germination with widespread colonization of the kernel and browning of the coleoptile; 4 = germination with reduced seedling development, complete colonization of the kernel, and lesions and abundant mold on the shoot, 5 = no germination due to complete rotting of the kernel.

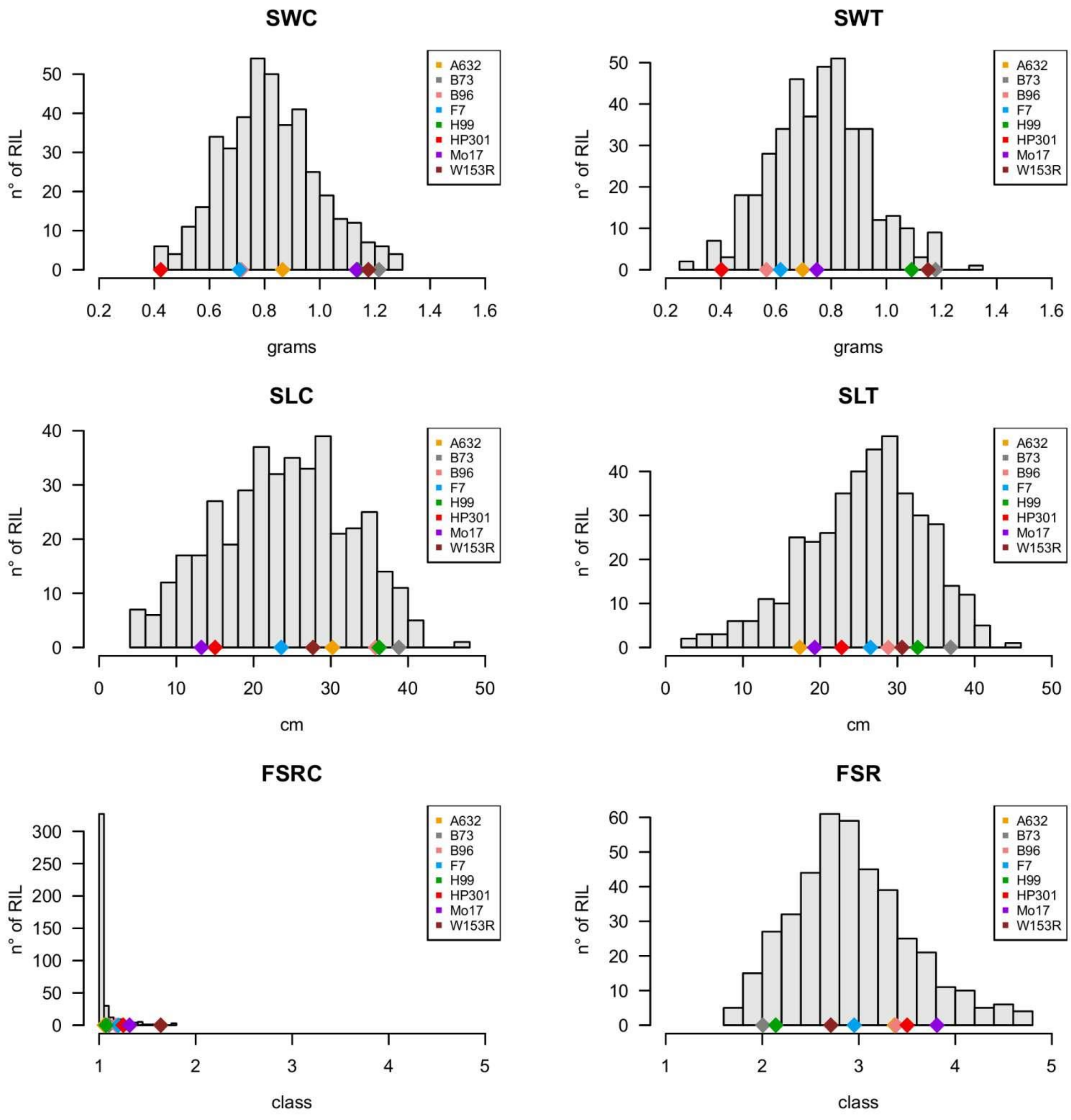

Figure S3. Distribution of phenotypic value of Fusarium seedling rot in control (FSRC) and in treated (FSR) rolled towel assays (RTAs), seedling length in control (SLC) and in treated (SLT) RTAs, and seedling weight in control (SWC) and in treated (SWT) RTAs. The color dots in histograms depict severity class of eight founder lines. Each founder names with its color is shown on the legend.

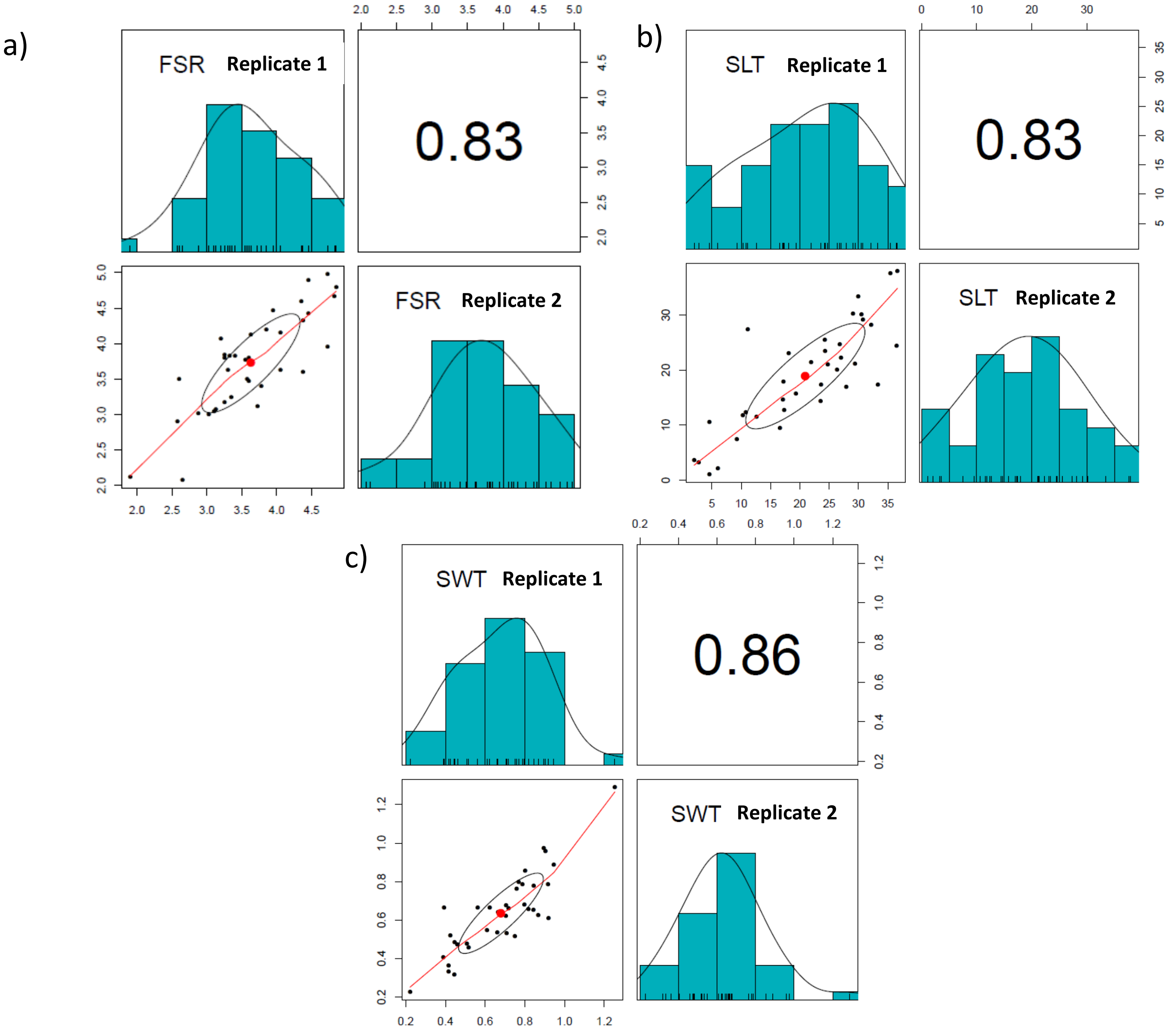

Figure S4. Correlation plot for replicated treated rolls. In each panel, an histogram of the distribution of the trait values is reported for the two replicas. The Pearson correlation coefficient is shown in the quadrant top right, and the relative scatterplot is shown bottom left with the corresponding trend line and correlation ellipse.

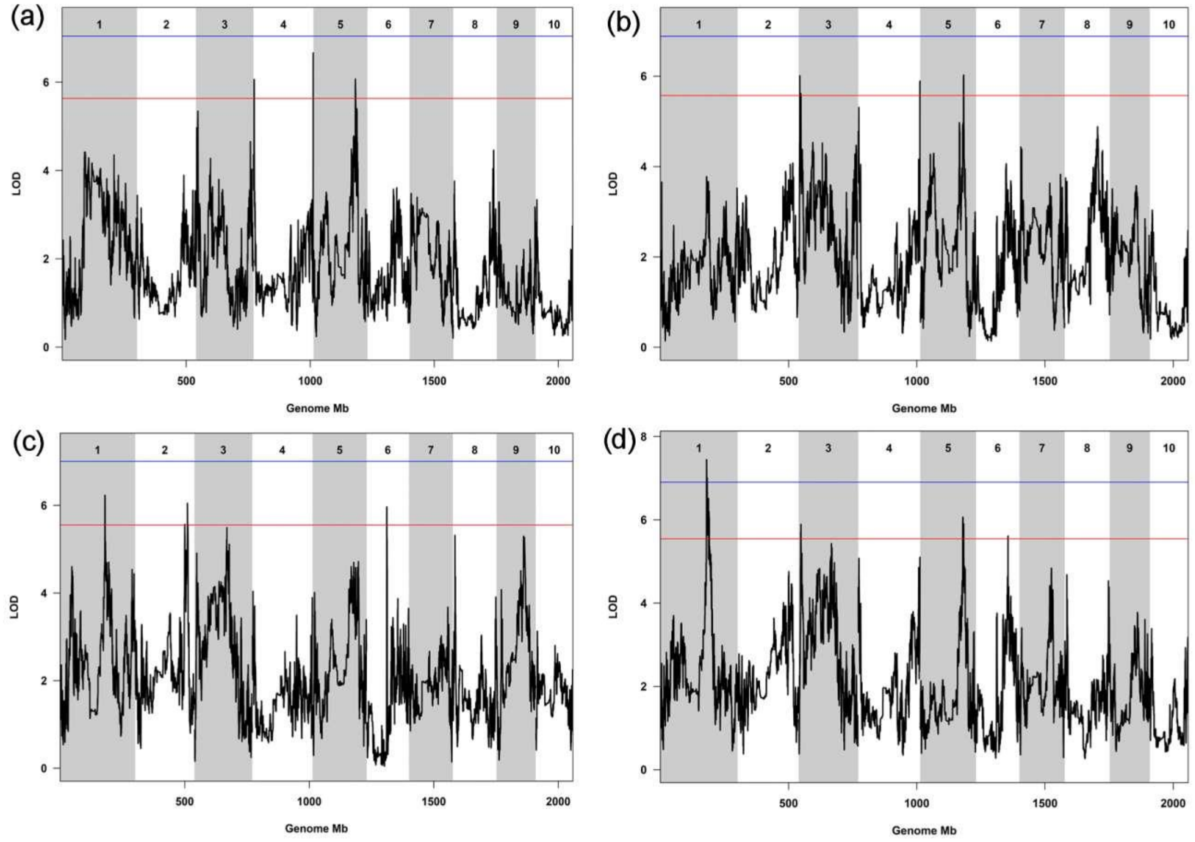

Figure S5. QTL scan for Fusarium seedling rot (FSR), seedling weight and seedling length. In panel **a**, QTL scan detects three QTL for FSR, two in chr 4 and one in chr 5. In panel **b**, QTL scan detects a unique QTL for SLT in chr 3. In panel **c**, QTL scan identified three QTL for SWC in chr 2 and 6. In panel **d**, QTL scan detects three QTL for SWT in chr 1, 3 and 6. Red and blue lines represent suggestive (P<0.63) and strong (P<0.1) thresholds, respectively.

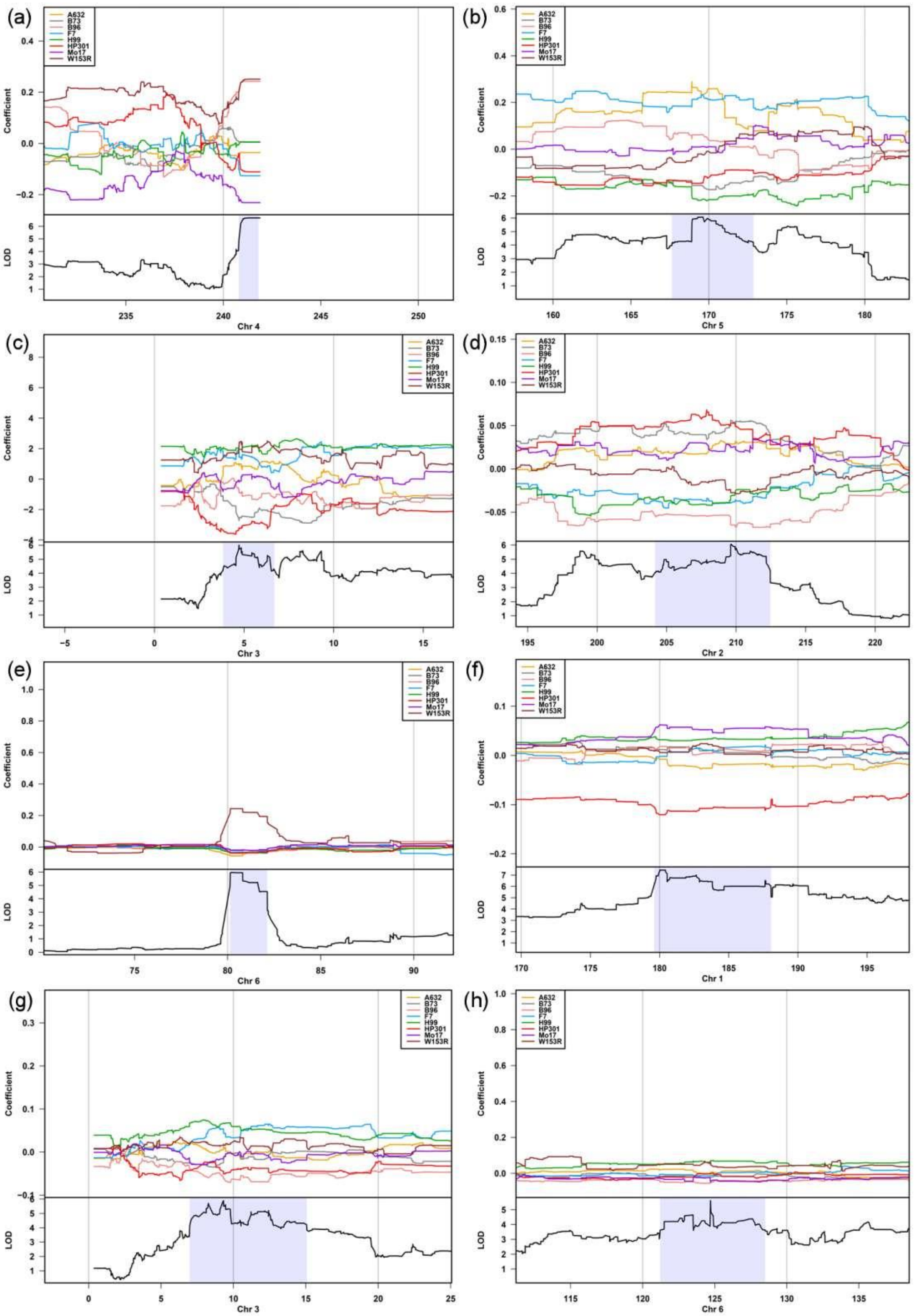

Figure S6. Founder allele effect estimation. The upper panel of each picture shows the founder contribution of each QTL and the bottom panel shows the QTL confidence interval in gray area. **a** qFSR4.2; **b** qFSR5.1; **c** qSLT3.1; **d** qSWC2.1 and qSWC2.2; **e** qSWC6.1; **f** qSWT1.1; **g** qSWT3.1; **h** qSWT6.1.

Table S1 Description of FSR classes.

Class	Description
1	Seed is well germinated and no visible sign of colonization
2	Seed is well germinated with little colonization on the kernel near the pedicel
3	Seed is germinated with ample colonization on the kernel and visible sign of browning on the coleoptile
4	Seed is germinated with reduced development, complete colonization of the kernel, and lesions and abundant mold on the shoot
5	Seed is not germinated due to complete rotting of the kernel

Table S2 The list of recombinant inbred line (Supplementary dataset).

Table S3 Phenotypic value of Fusarium seed rot in control (FRSC) and in treated (FSR) rolled towel assays (RTAs), seedling length in control (SLC) and in treated (SLT) RTAs, seedling weight in control (SWC) and in treated (SWT) RTAs.

Trait	Founder lines				Recombinant Inbred Lines			
	Min	Max	Mean	SD	Min	Max	Mean	SD
FSRC	1	1.6	1.2	0.18	1	1.7	1	0.1
FSR	2	3.8	2.9	0.65	1.6	4.7	2.9	0.6
SWC (gram)	0.42	1.2	0.9	0.28	0.4	1.2	0.8	0.1
SWT (gram)	0.4	1.2	0.8	0.29	0.2	1.3	0.7	0.1
SLC (cm)	13.2	38.8	27.6	9.67	4.1	47.2	23.6	8.6
SLT (cm)	17.5	37.2	26.9	6.75	2.7	45.7	25.9	7.6

Table S4 Phenotypic data of the replicated 36 recombinant inbred lines (Supplementary dataset).

Table S5 Protein coding genes detected in differential expression analysis (Supplementary dataset).

Table S6 Protein coding genes detected in association mapping (Supplementary dataset).
